# Supplementary figures and images for: Idiosyncratic liver pigment alterations of five frog species in response to contrasting land use patterns in the Brazilian Cerrado
Source: PeerJ. 2020 Aug 26;8:e9751. doi: 10.7717/peerj.9751 (PMC7456255; doi:10.7717/peerj.9751)

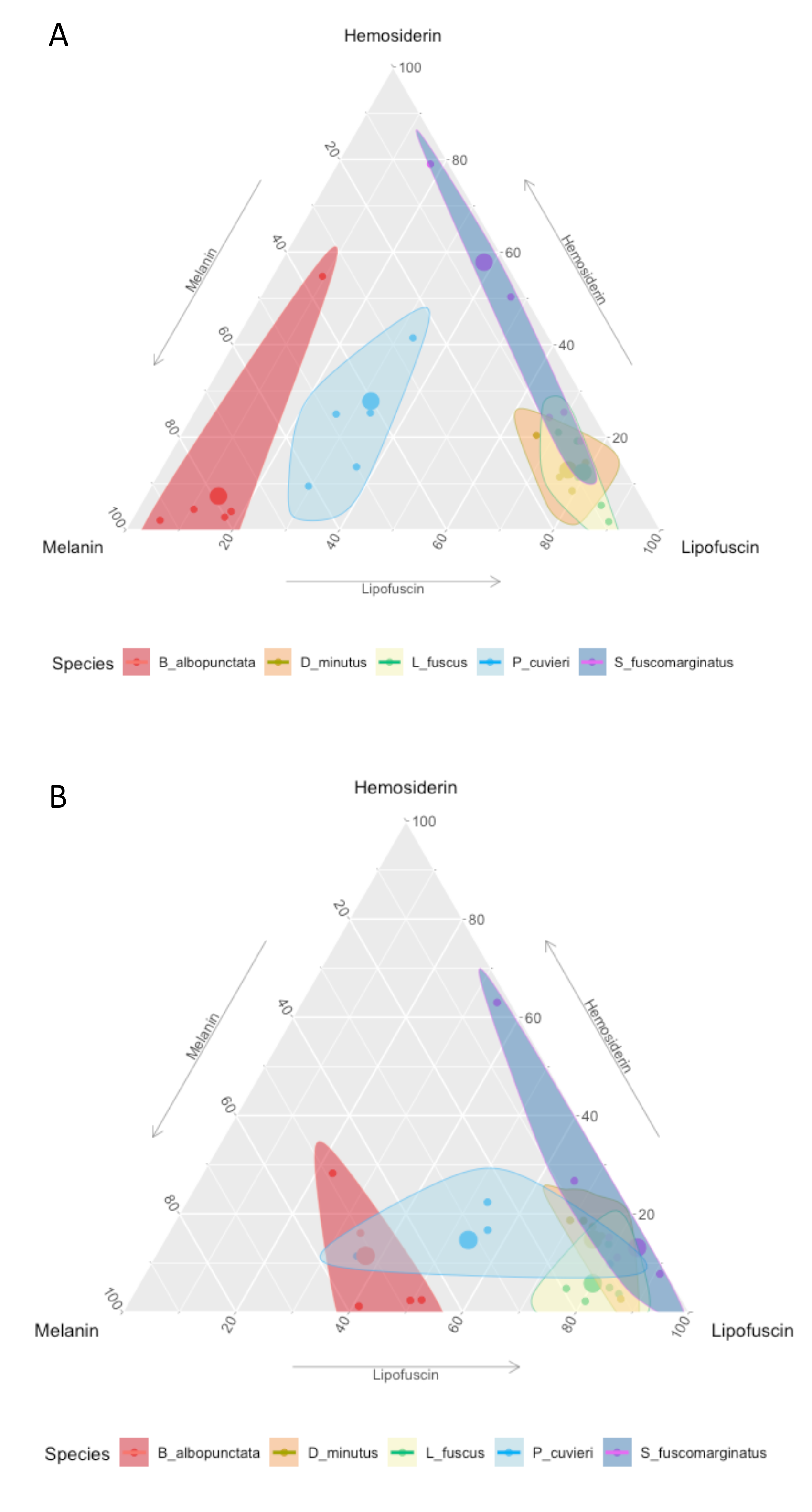

Supplement: Supplemental Information 2 — (A) Protected Area and (B) agricultural region. Small points represent individual measurements, while the large dot represents the mean of each substance for each species. [file peerj-08-9751-s002.png]
